# Alterations of Regional Spontaneous Brain Activity and Gray Matter Volume in the Blind

**DOI:** 10.1155/2015/141950

**Published:** 2015-10-19

**Authors:** Aili Jiang, Jing Tian, Rui Li, Yong Liu, Tianzi Jiang, Wen Qin, Chunshui Yu

**Affiliations:** ^1^Department of Radiology and Tianjin Key Laboratory of Functional Imaging, Tianjin Medical University General Hospital, Tianjin 300052, China; ^2^Brainnetome Center, Institute of Automation, Chinese Academy of Sciences, Beijing 100190, China

## Abstract

Visual deprivation can induce alterations of regional spontaneous brain activity (RSBA). However, the effects of onset age of blindness on the RSBA and the association between the alterations of RSBA and brain structure are still unclear in the blind. In this study, we performed resting-state functional and structural magnetic resonance imaging on 50 sighted controls and 91 blind subjects (20 congenitally blind, 27 early blind, and 44 late blind individuals). Compared with the sighted control, we identified increased RSBA in the blind in primary and high-level visual areas and decreased RSBA in brain regions which are ascribed to sensorimotor and salience networks. In contrast, blind subjects exhibited significantly decreased gray matter volume (GMV) in the visual areas, while they exhibited significantly increased GMV in the sensorimotor areas. Moreover, the onset age of blindness was negatively correlated with the GMV of visual areas in blind subjects, whereas it exerted complex influences on the RSBA. Finally, significant negative correlations were shown between RSBA and GMV values. Our results demonstrated system-dependent, inverse alterations in RSBA and GMV after visual deprivation. Furthermore, the onset age of blindness has different effects on the reorganizations in RSBA and GMV.

## 1. Introduction

Visual deprivation can induce a series of structural and functional reorganizations of the brain to better adapt to external environments with the remaining modality, especially in individuals with visual deprivation at an early developmental age. For example, early visual deprivation can remold the visual areas to cross-modal process signals from nonvisual modalities, such as tactile [1, 2], auditory [3–5], and even olfactory processes [6]. Furthermore, a large number of studies have demonstrated that the visual areas have increased baseline metabolism and blood flow [7–9], decreased anatomical network efficiency [10], and altered resting-state functional connectivity [11] in congenitally blind (CB) or early blind (EB) subjects. Although impaired visual areas have also been reported, limited studies have demonstrated that late blind (LB) individuals had different alteration patterns with CB/EB individuals, including the cross-modal activity [4, 12, 13], baseline glucose metabolism [9], cortical thickness [8, 14, 15], gray matter content [16], anatomical connectivity [10], white matter integrity [17], and functional connectivity density (FCD) [18]. These findings indicate that visual deprivation at different developmental stages may influence both functional and structural organizations of the brain [19]. However, it is unclear if the age of onset of blindness influences the regional spontaneous brain activity.

Spontaneous neuronal activity can be identified by low-frequency fluctuations in the blood oxygen level dependent (BOLD) signal of functional magnetic resonance imaging (fMRI) under resting-state [20]. As a data-driven resting-state fMRI technique, regional homogeneity (ReHo) measures the functional coherence of BOLD fluctuations of a given voxel with its nearest neighbors. It can be used to evaluate the regional spontaneous brain activities based on the hypothesis that significant brain activities would more likely occur in clusters than in a single voxel [21]. This technique has been successfully applied to investigate the spontaneous brain functional organization in healthy subjects [22] and to elucidate the neural pathological mechanisms of a variety of neurological and psychiatric diseases [23, 24]. Regarding the functional alterations of regional spontaneous brain activity after visual deprivation, one recent study demonstrated that EB had increased ReHo in the primary and higher visual areas, which indicates an abnormal cortical development and/or experience-dependent plasticity secondary to early visual deprivation [25]. However, the effects of visual deprivation during different developmental periods on the regional spontaneous brain activity are rarely known.

Another concerning issue is the potential associations between the alterations of regional spontaneous brain activity and brain structural organization in blind individuals. Although abundant studies have demonstrated that the visual-deprived brain experienced both functional and structural alterations (as described in the first paragraph), the relationships between them have not been clearly identified. In a recent study, Anurova et al. combined the measurements of cortical thickness and functional activation evoked by attention-demanding auditory tasks. They identified a significant negative correlation between the cortical thickness and cortical activation in the visual areas in the EB [3]. This finding indicates that pruning of exuberant connections during early development increases the selectivity and effectiveness of synaptic activity and therefore leads to stronger activation, which provides a structural basis for cross-modal occipital activation. Thus, clarification of the association between the alterations of regional spontaneous brain activity and brain structural organization may help our understanding of the neural mechanisms of functional alterations during the resting-state.

In this study, we combined voxel-wise ReHo and gray matter volume (GMV) approaches to investigate the alterations of the regional spontaneous brain activities and brain structures and their relationships in blind individuals. The functional and structural alterations of the brain after visual deprivation comprise the synthetic interactions among developmental, plastic, and degenerative mechanisms [14]. The developmental mechanisms play important roles in both the CB and EB; and the capacity for cross-modal plasticity is substantially stronger in the CB/EB than the LB, whereas degenerative mechanisms exert their effects on all blind subjects. Thus, we first hypothesized that visual deprivation during different developmental periods may induce diverse patterns of alterations in ReHo and GMV in the visual areas. Specifically, because earlier studies have demonstrated that the CB/EB experienced stronger increases in cross-modal activity [4, 26] and functional connectivity density [18], while they experienced smaller decreases in white matter integrity [17] than the LB in the visual pathway, we predicted that blind subjects who lost their vision at an earlier developmental age would experience a greater increase in ReHo but less degenerative-induced atrophy in the visual areas. Second, because early studies have shown increased white matter integrity in the corticospinal tract [17] and decreased long-range functional connectivity density in the primary sensorimotor area [18] in both CB and LB, we predicted significant alterations of both ReHo and GMV in the spared sensory areas in all blind groups regardless of the onset of age of blindness. Finally, we expected significant correlations between ReHo and GMV in these areas in the blind, which may help to elucidate the neural mechanisms of functional alteration during the resting-state.

## 2. Materials and Methods

### 2.1. Subjects

The subjects are ninety-one right-handed blind subjects (63 males and 28 females, age range from 20 to 45 years) and 50 sighted controls (SC) (33 males and 17 females, age range from 19 to 44 years) with no history of neurological and psychiatric problems; the excluded criteria included nonright handedness, different onset ages of blindness between the left and right eyes, and low-quality data. The blind subjects were further divided into three subgroups, including 20 congenital blind (CB), 27 early blind (EB) (age of onset ≤ 12 years), and 44 late blind (LB) (age of onset > 12 years). None of the CB subjects had a history of pattern vision or memory of visual experience, and none of the acquired blind subjects had experienced pattern vision after visual deprivation. All the SC subjects had normal vision with corrected visual acuity higher than 0.8 (decimal record) [27], and subjects with history of severe ophthalmic diseases, such as glaucoma, cataract, and retinal detachment, were excluded from the study by questionnaire. The demographic and behavioral data of these subjects are shown in Table 1. The study was approved by the Ethics Committee of Tianjin Medical University, and all subjects provided written informed consent.

### 2.2. MRI Data Acquisition

The structural and functional MRI data were acquired using a 3.0-Tesla MR scanner (Magnetom Trio, Siemens, Erlangen, Germany). The subjects' heads were fixed using foam pads to minimize head motion, and earplugs were used to reduce the scanning noise. The resting-state fMRI data were obtained using a gradient echo single-shot echo planar imaging (GRE-SS-EPI) sequence with the following parameters: repetition time (TR) = 2000 ms, echo time (TE) = 30 ms, flip angle (FA) = 90°, matrix = 64 × 64, field of view (FOV) = 220 mm × 220 mm, slice thickness/gap = 3/1 mm with 32 axial slices, 180 volumes (time points), and a parallel acquisition technique with an acceleration factor of 2. During fMRI scans, all subjects were instructed to keep their eyes closed, relax, do not move, think of nothing in particular, and stay awake. After the fMRI scan, the fMRI images and subjects' conditions were checked to confirm whether they satisfied the requirements; if not, the fMRI data were abandoned and scanned again. Structural images were acquired using a 3D magnetization-prepared rapid-acquisition gradient echo sequence with the following parameters: TR/TE/inversion time = 2000/2.6/900 ms, FA = 9°, matrix = 256 × 224, FOV = 256 mm × 224 mm, and 176 continuous sagittal slices with a 1 mm thickness.

### 2.3. Data Processing

#### 2.3.1. Creating Customized Templates

Data processing was performed using a self-developed software based on Matlab v2009 (MathWorks, Inc., Natick, Massachusetts, USA) and SPM8 (http://www.fil.ion.ucl.ac.uk/spm). First, the structural MR images for each subject were segmented into gray matter, white matter, and cerebrospinal fluid using the standard unified segmentation model in SPM8. Then the 3 tissue probabilistic maps (TPMs) of each subject were affinely coregistered with a standard TPM template implemented in SPM8, separately. After that, the 3 types of coarsely coregistered TPMs of all the blind and sighted subjects (112 cases) entered into a nonlinear image coregistration procedure using diffeomorphic anatomical registration through the exponentiated Lie algebra (DARTEL) technique [28], which involves iteratively matching all the selected images to a template generated from their own mean. This step generated 6 sets of tissue templates from coarse to refined contours. These customized DARTEL templates were used for following normalization of the structural and functional MRI data.

#### 2.3.2. VBM Analysis

After an initial affine coregistration, the gray matter probabilistic map was nonlinearly warped into the customized DARTEL templates and was resliced with a resolution of 1.5 × 1.5 × 1.5 mm^3^. The GMV of each voxel was obtained by multiplying the gray matter concentration map by the nonlinear determinants derived from the spatial warping step, which in effect represents the relative GMV after removing the confounding effect of variance in individual brain sizes. Finally, the GMV map was smoothed with an isotropic Gaussian kernel of 6 × 6 × 6 mm^3^ FWHM.

#### 2.3.3. ReHo Analysis

The first 10 volumes of each functional time series were discarded because of the instability of the initial MRI signals caused by incomplete T1 relaxation and to allow the subjects to adapt to the scanning environments. The remaining 170 volumes were subsequently forwarded to a series of preprocessing steps, including slice timing (corrected for acquisition time delay between different slices), rigid realignment (corrected for intervolume head motion; all subjects' fMRI data were within the head motion thresholds for a maximum translational displacement lower than 2 mm or a maximum rotational displacement lower than 2.0°), nuisance regression (including six rigid motion parameters and their first derivatives, the mean BOLD signal of cerebrospinal fluid and white matter, and the spike time points with a mean framewise displacement higher than 0.5), band-pass filtering (0.01 to 0.08 Hz), and normalization (two-step coregistration method: first, the mean fMRI image generated at the realignment step was affinely coregistered with individual structural images; then each filtered functional image was spatially normalized into the customized DARTEL space using the deformation determinants derived from structural normalization step and was resampled into a 3 mm cubic voxel).

The ReHo map for each subject was calculated using REST software (http://www.restfmri.net/). ReHo measures the Kendall correlation coefficient of a given voxel and those of its direct neighbor voxels (27 voxels) in a voxel-wise manner [21]. A higher ReHo value of a voxel indicates that the regional activity of this voxel is more similar to its neighbors. We also calculated the ReHo values with different neighboring strategies (7 voxels and 19 voxels, resp.) to explore if neighboring strategies would influence our result [21]. The calculated ReHo values for each subject were further scaled by the mean ReHo of the whole brain to reduce the effect of individual variation. Finally, the scaled ReHo maps underwent spatial smoothing using a Gaussian kernel with full width half maximum (FWHM) of 6 × 6 × 6 mm^3^.

### 2.4. Statistical Analysis

One-way analysis of variance (ANOVA) was conducted in a voxel-wise manner to investigate the intergroup differences in ReHo and GMV values among the four groups with age and gender as nuisance covariates within the cerebral gray matter mask. The statistical *F* map was corrected for multiple comparisons using a Monte Carlo simulation method at the cluster level (Alphasim algorithm, voxel-wise *P* < 0.01, iteration 5000 times, and corrected cluster-wise *P* < 0.05), resulting in a corrected cluster size of 34 voxels for ReHo analyses and 260 voxels for GMV analyses. The clusters with statistical significance in ReHo and GMV values were defined as the regions of interest (ROIs). The voxels within the 9 mm radius sphere centering the peak (also second peak when the cluster occupied multiple brain regions) of each cluster, as well as surviving under the statistical threshold, were assigned to a specific ROI. The second peak was defined as the peak voxel that can be separated from the cluster containing the highest peak when we increase the statistic threshold and had distance at least 30 mm away from the highest peak. Then the mean ReHo and GMV values of each ROI in each subject were extracted. The associations between the ReHo/GMV and the age of onset of blindness were analyzed using partial correlation analyses that are controlled for gender and age effects. Finally, the associations between the ReHo and GMV values in the brain regions that exhibited altered ReHo after blindness were analyzed using partial correlation analyses controlled for age and gender effects (*P* < 0.05, uncorrected).

## 3. Results

### 3.1. Demographic Information

As shown in Table 1, there were no significant differences in age (one-way ANOVA, *F* = 2.07, and *P* = 0.107) or gender (chi-square test, *χ* ^2^ = 0.63, and *P* = 0.889) among the 4 groups. There was also no significant group difference in framewise displacement (one-way ANOVA, *F* = 0.425, and *P* = 0.716), which indicates that gender, age, and head motion might not interpret the possible differences in ReHo among the CB, EB, LB, and SC.

### 3.2. The Influence of Number of Neighboring Voxels on the ReHo Measurements

To clarify if the number of neighboring voxels would influence the measurement of ReHo and group comparison in our study, we calculated the ReHo value using 7, 19, and 27 voxels and compared the intergroup differences in ReHo using one-way ANOVA (*P* < 0.05, corrected at the cluster level), respectively. We found a lower noise and higher contrast in ReHo map with higher voxel size (see Supplementary Figure S1 in Supplementary Material available online at http://dx.doi.org/10.1155/2015/141950). Furthermore, we compared the intergroup differences in ReHo values that were calculated using different neighboring voxels using one-way ANOVA. As shown in Supplementary Figure S2, the *F* distributions of intergroup differences were similar among the three datasets, and the dataset using 19 and 27 was more statistically significant in the middle cingulate cortex (MCC) than that using 7 voxels. Based on the above evidences, we chose the ReHo of 27 neighboring voxels as the functional metric in the following analyses.

### 3.3. ReHo Alterations in the Blind

**Figure 1:**
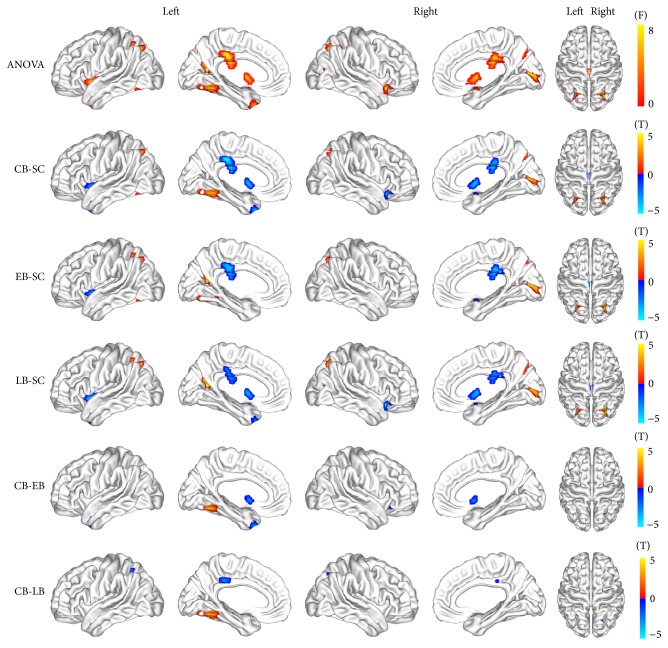
Brain regions with altered regional homogeneity in blind individuals. One-way analysis of variance was used for intergroup comparison, which was controlled for age and gender effects (*P* < 0.05, corrected using Monte Carlo simulation).

Compared with the sighted subjects (*P* < 0.05, corrected at the cluster level), the blind subjects generally had significantly increased ReHo in the primary and higher visual pathways, including the left fusiform gyrus (FG), left superior parietal lobule (SPL), left parietooccipital sulcus (POS), right calcarine sulcus (CalS), right middle occipital gyrus (MOG), and right superior of occipital gyrus (SOG), and decreased ReHo in the sensorimotor and salience networks, including the bilateral putamen, middle cingulate cortex (MCC), bilateral anterior insula (aINS), and bilateral temporal pole (TP). In the left FG, the CB demonstrated increased ReHo compared to EB, LB, and SC. Within the blind subjects, the CB exhibited lower ReHo in the left SPL and MCC than the LB and lower ReHo in the bilateral putamen, right aINS, and right TP than the EB (Figure 1).

### 3.4. GMV Alterations in the Blind

**Figure 2:**
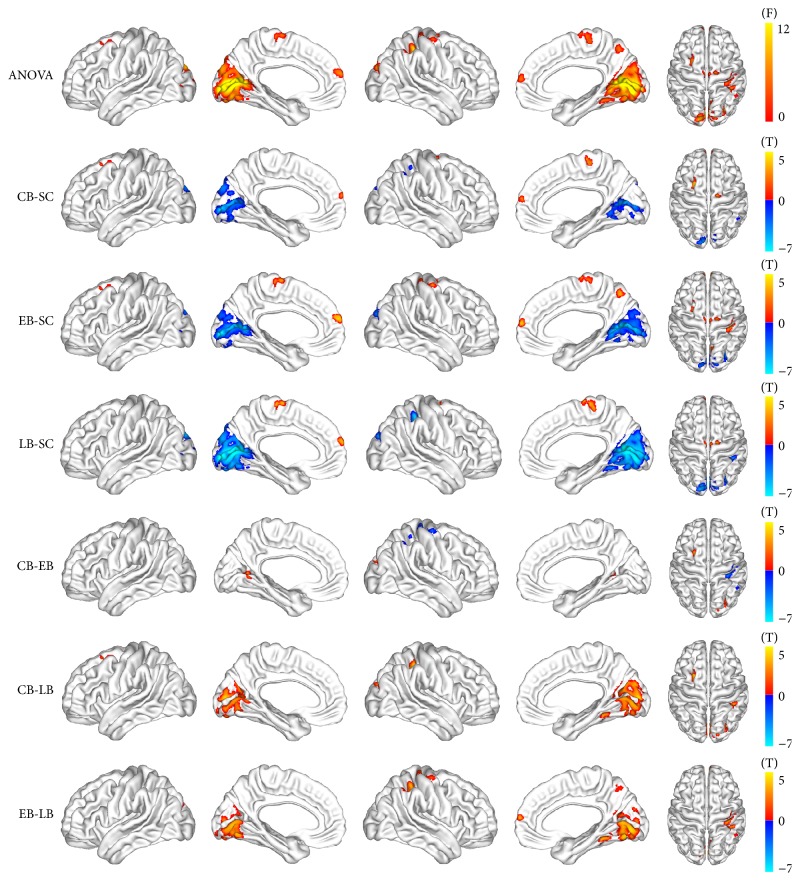
Brain regions with altered gray matter volume in blind individuals. One-way analysis of variance was used for intergroup comparison, which was controlled for age and gender effects (*P* < 0.05, corrected using Monte Carlo simulation).

A voxel-wise ANOVA indicated that the intergroup difference in GMV was mainly located in the visual and sensorimotor cortices (*P* < 0.05, corrected at the cluster level). Specifically, decreased GMV of the blind was identified in the primary and higher visual pathways relative to the SC, including the bilateral CalS, cuneus, lingual gyrus, FG, and SOG, and right intraparietal area. Furthermore, the older the age of onset of blindness is, the broader the atrophy of the visual cortex could be identified. Increased GMV was also identified in the right postcentral gyrus (PostCG) and precuneus (PreCu) of the EB, the left superior frontal gyrus (SFG) of the CB and EB, and the precentral gyrus (PreCG), medial prefrontal cortex (MPFC), and paracentral gyrus (ParaCG) of all blind groups. Within the blind subjects, the CB and EB generally had higher GMV in the visual associate areas compared with the LB. The EB had higher GMV than the CB and LB in the right PostCG, and the CB had higher GMV than the LB in the left SFG (Figure 2).

### 3.5. Correlation Analyses

**Figure 3:**
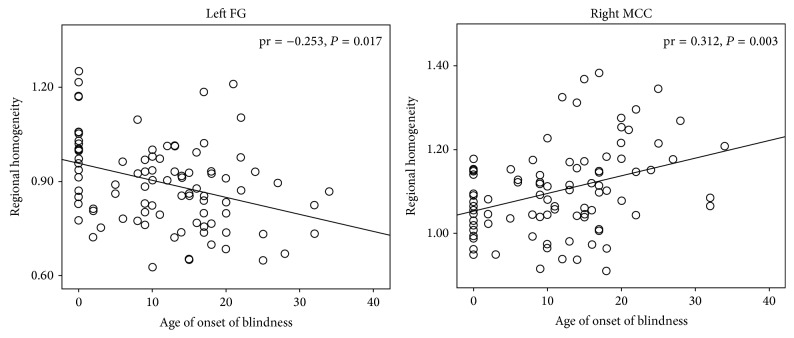
Correlation between the regional homogeneity (ReHo) and age of onset of blindness. Partial correlation analysis was performed, which was controlled for age and gender effects (*P* < 0.05, uncorrected). FG: fusiform gyrus; MCC: middle cingulate cortex.

**Figure 4:**
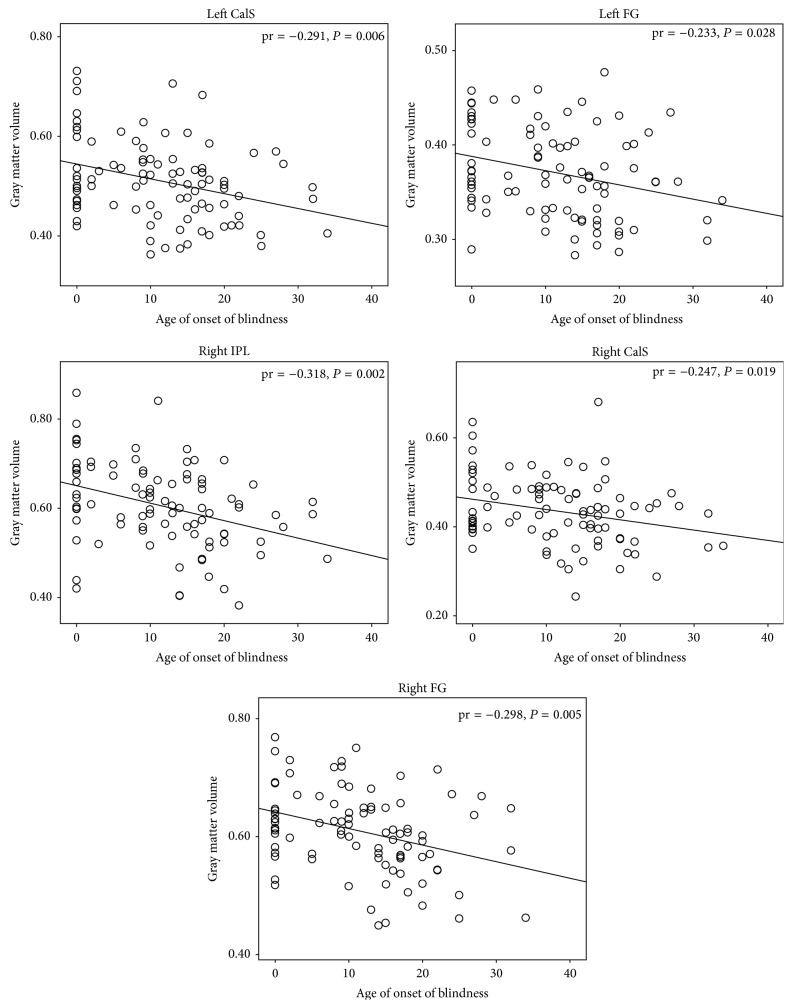
Correlation between the gray matter volume (GMV) and age of onset of blindness. Partial correlation analysis was performed, which was controlled for age and gender effects (*P* < 0.05, uncorrected). CalS: calcarine sulcus; FG: fusiform gyrus; IPL: inferior parietal lobe.

**Figure 5:**
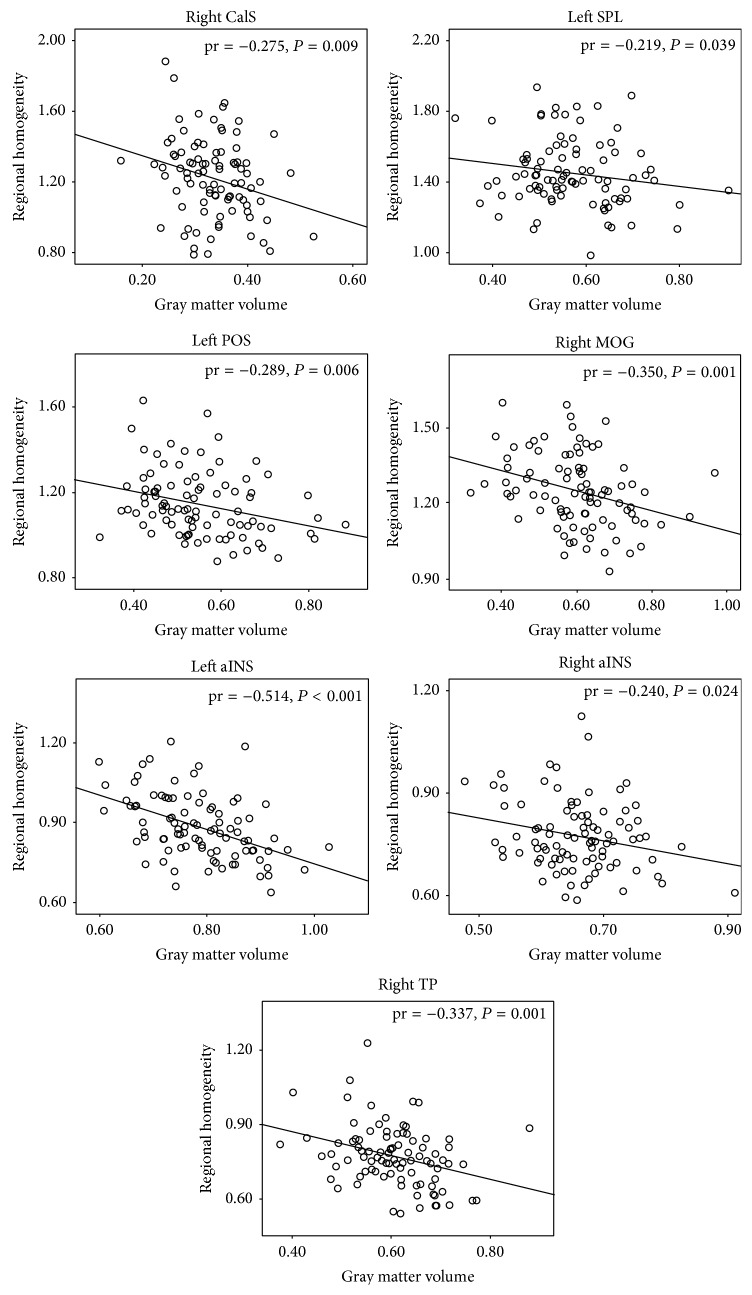
Correlation between the regional homogeneity (ReHo) and gray matter volume (GMV) in blind individuals. Partial correlation analysis was performed, which was controlled for age and gender effects (*P* < 0.05, uncorrected). aINS: anterior insula; CalS: calcarine sulcus; MOG: middle occipital gyrus; POS: parietooccipital sulcus; SPL: superior parietal lobule; TP: temporal pole.

Partial correlation analyses indicated that the age of onset of blindness was negatively correlated with the ReHo of the left FG but positively correlated with the MCC (*P* < 0.05, uncorrected) (Figure 3); moreover, the age of onset of blindness was negatively correlated with the GMV of the bilateral CalS, bilateral FG, and right IPL (*P* < 0.05, uncorrected) (Figure 4). In the brain regions that exhibited intergroup differences in ReHo, significant negative correlations between the ReHo and GMV values were identified in the left POS, left SPL, right CalS, right MOG, right TP, and bilateral aINS (*P* < 0.05, uncorrected) (Figure 5).

## 4. Discussion

In this study, we identified system-dependent inverse alterations in the regional spontaneous brain activity and gray matter volume in blind individuals: in the primary and higher visual areas, increased ReHo and decreased GMV were identified in the blind subjects, whereas in the sensorimotor related areas, decreased ReHo and increased GMV were identified. Furthermore, significant negative correlations between the ReHo and GMV values were identified in most brain regions with altered ReHo in the blind. Finally, the age of onset of blindness had different effects on the alteration of brain function and structure. Our findings provided additional information regarding the alteration patterns of the regional spontaneous brain activity and their structural bases after visual deprivation at different developmental stages.

### 4.1. System-Dependent Alteration of the Regional Spontaneous Brain Activity and Structure in Blind Individuals

The pattern of increased ReHo of the visual cortex in the CB/EB was similar to a recent study of our groups [25]. We also identified decreased GMV in the visual cortex. Combined with earlier findings of increased baseline glucose metabolism [7–9] and cortical thickness [8, 15] in the early occipital areas in this population, a potential relationship may exist between the alterations in functional and structural organizations in the deprived visual cortex. The first possible explanation is that the increased ReHo of the CB in the visual cortex reflects the retention of exuberant connections within the occipital cortex that resulted from developmental interruption. The development of the human visual cortex is characterized by an initial overproduction of synaptic connections with a maximum synaptic density at approximately 8 months, followed by the pruning of inactive synapses to reach “adult” levels at approximately 11 years [29]. Although the first phase of synaptogenesis is independent of retinal input [30], synaptic pruning is driven by visual experience [31]. Therefore, the exuberant connections caused by failed refinement can partially explain the increased ReHo, metabolism, and cortical thickness in the visual cortex. However, the “pruning interruption” hypothesis cannot solely explain the findings that a large number of concurrent reports have demonstrated that early deprived visual areas are involved in the cross-modal processing of tactile [1, 2, 32], auditory [3–5], and even olfactory tasks [6]. Thus, other mechanisms, including the establishment of new connections (rewiring theory) or reinforcement of existing connections (unmasking theory) [8], may also contribute to the increased regional brain activity in the CB/EB. Finally, decreased GMV was identified in the visual cortex in the CB/EB, which is consistent with previous reports [16, 33]. In several earlier studies, thickened cortical thickness [3, 8, 14, 15, 33] and reduced cortical surface area [14, 33] were found in the occipital cortex of the CB and EB. Because the GMV is determined by both cortical thickness and cortical surface area, the reduced GMV in the visual cortex in the CB and EB might be caused by overly reduced surface area after visual deprivation, which masks the enhancing effect caused by cortical thickness. Thus, the increased ReHo might also represent a compensation for disuse-induced atrophy of the surface area of visual cortex.

Similar to the CB/EB, we also identified significantly increased ReHo of the visual cortex in the LB. To our knowledge, this is the first study to focus on the regional spontaneous brain activity in late blind subjects. This finding was consistent, in part, with a recent study that demonstrated increased functional connectivity density along the dorsal and ventral visual pathways in the LB [18]. In contrast to the CB/EB, the LB demonstrated different brain alteration patterns, including weaker cross-modal activity [4, 12, 13], higher baseline glucose metabolism [9], and thinner cortical thickness of the visual cortex [8, 14, 15]. Thus, although the LB and the CB/EB had similar increases in ReHo in the visual cortex, they might represent different neural mechanisms. The connections of visual cortex have already finished normal refinement for visual processing prior to visual deprivation in the LB, so developmental factors, such as “rewiring” or “pruning interruption,” are not possible in them. Thus, the increased ReHo in the LB highly indicates the synthetic influence of plastic and degenerative factors. The plastic factor strengthened the existing intra- and intercortical connections of the occipital cortex, whereas the degenerative factor destroyed the existing connections. These synthetic effects can explain why increased cross-modal activation [4, 34] and functional synchronization [18] are accompanied by damaged gray and white matter structures of the visual cortex in the LB [16, 17].

It should be noted that we identified an increased ReHo in the primary visual cortex in all blind subjects, whereas most previous studies have demonstrated a decreased FC (functional connectivity) of the same area [11, 12]. This disparity may be caused by the different roles of the ReHo and functional connectivity in delineating the intrinsic functional organization: ReHo specifically reflects the synergistic action of neighboring clustered neurons, whereas FC represents the intrinsic functional synchronization of the visual cortex with remote areas, such as the sensorimotor and auditory cortices. The increased ReHo in the early visual cortex and decreased FC (or FCD) between the early visual and nonvisual sensory cortices suggest that local efficiency of the early visual cortex may be strengthened to compensate for a decreased long-range transfer efficiency between visual and other sensory modalities.

With the exception of the increased ReHo in the visual cortex in the blind, we also identified decreased ReHo in the putamen and MCC, which are ascribed to the sensorimotor network (SMN). In contrast, increased GMV was also identified in the SMN (including the PostCG, PreCG, and ParaCG). The increased GMV in the SMN was consistent with previous studies that demonstrated increased white matter volume [35] in the sensorimotor system of the EB and increased white matter integrity of corticospinal tracts in both CB and LB [17]. The MCC exhibits extensive FC with brain regions that belongs to the SMN and has direct connections with the spinal cord [36], which is consistent with its function for motor and pain processes [37]. The putamen is an important hub in the complex extrapyramidal motor system. It receives inputs from many cortical areas and subcortical nuclei and principally projects to the prefrontal, premotor, and supplementary motor areas. It plays roles in motor planning, initiation, and regulation [38]. The loss of sight in the blind may require more sensorimotor and motor practice, such as Braille reading, spatial localization, and object recognition, which might induce the plasticity of the SMN, as indicated by increased GMV, white matter volume [35], and white matter integrity [17] in related structures. Thus, the decreased ReHo in the MCC and putamen may be related to experience-dependent plasticity by extensive use of the limbs in the blind.

We also identified decreased ReHo in the anterior insula and temporal pole, which are ascribed to the salience network (SN). As a core hub of the salience network, the anterior insula serves to identify most relevant and salient stimuli from visual, auditory, and other sensory inputs and then initiates control signals to the central executive network that mediate attention, working memory, and other higher order cognitive processes while it disengages the default mode network [39]. The loss of sight makes blind subjects depend more on nonvisual inputs to efficiently interact with the environment. It is interesting to note that Wang et al. recently reported an increase in FC within the salience network and between the salience and frontoparietal networks in the CB [40]. In the present study, we also identified the alteration of the regional spontaneous brain activity within the salience network. The alteration of regional brain activity and FC in the salience network might reflect enhanced attentional ability to identify salient stimuli from the auditory, tactile, and other sensory inputs. Superior attentional performance has been reported in the CB/EB [41–43]. However, the functional roles of decreased ReHo of the salience network, the relationships between increased FC and decreased ReHo, and the relationships between spontaneous functional alteration and task-evoked activation of this network require future clarification.

### 4.2. Inverse Association between Regional Spontaneous Brain Activity and Gray Matter Volume in the Blind

One of the main findings of the present study is an inverse relationship between the alterations of ReHo and GMV in blind individuals: the increases in ReHo were accompanied by decreases in GMV of visual areas, and the decrease in ReHo was accompanied by an increase in GMV of the SMN. Furthermore, negative correlations between the ReHo and GMV were identified in these visual and SMN areas. This finding was in line with a recent study that demonstrated a negative correlation between the magnitude of activation and cortical thickness of the visual cortex in EB subjects [3]. The inverse relationships between the ReHo and GMV suggest a balance between the structural and functional alterations of the brain after visual deprivation. On one hand, visual loss directly induces structural impairment of both white matter [44–46] and gray matter [15, 45] in the visual cortex. The remaining neurons may be overloaded to process the information from other modalities, which leads to an increase in neuronal activity by nonvisual stimuli [4, 26] or internally top-down signals [1, 47], and the activity status of remnant visual neurons persists at the resting-state, as indicated by increased ReHo and baseline metabolisms [7–9]. On the other hand, to adapt to the external environment without visual signals, long-term visual loss indirectly drives the experience-dependent plasticity of nonvisual structures [17]. Thus, the decreased ReHo may reflect the increased efficiency in processing nonvisual signals in these regions, which require a smaller number of neurons or lower threshold to handle the same tasks. However, this assumption should be verified by introducing the behavior performance associations.

### 4.3. Effects of Age of Onset of Blindness on the Regional Spontaneous Brain Activity and Gray Matter Volume

In this study, we demonstrated that the age of onset of blindness had a complex influence on the ReHo values of the brain. Specifically, in the left FG, the CB had higher ReHo than the EB and the LB; in the SOG, TP, putamen, and MCC, the CB generally had lower ReHo than the blind individuals who lost sight at older ages. Furthermore, the age of onset of blindness was negatively correlated with the ReHo of the left FG but positively correlated with the MCC. These findings were partially consistent with previous studies that demonstrated different alteration patterns of brain in blind individuals with lost sight at different developmental stages, including the cross-modal activity [4, 12, 13], baseline glucose metabolism [9], cortical thickness [8, 15], gray matter content [16], anatomical connectivity [10], white matter integrity [17], and FCD [18]. Our findings indicate that visual experience plays an important role in reshaping the regional spontaneous brain activity. In the ventral visual stream (as demonstrated by the left FG), only the CB (but not the EB or LB) exhibited increased ReHo compared to the SC, which was supported by a recent study that identified stronger FCD in the ventral stream of the CB relative to the LB [18]. These findings indicate that visual experience during the sensitive period is critical to normal development of the synchronization of regional clustered neurons in the ventral stream [48]. In the dorsal visual stream (as revealed by right SOG), although all blind individuals exhibited increased ReHo compared with the SC, a lower amplitude of increment was identified in the blind individuals with earlier sight lost. This finding indicates that developmental factors may not be a major determinant in reshaping these areas, which is supported by studies that demonstrate the dorsal visual stream matures more earlier than the ventral one [49, 50]. Instead, experience-dependent plasticity and degenerative factor may exert effects. Finally, in the nonvisual areas (such as the MCC and putamen), the CB exhibited a more extended decrement in ReHo than the EB and the LB compared with the SC. Because the development of these areas is independent of visual experience and, to our knowledge, there is no report of degenerative alterations of these areas after visual deprivation, experience-dependent plasticity may be the major factor that accounts for it. However, the functional roles of the alteration in regional brain activity remain unknown and require further clarification.

In contrast to the ReHo, the blind subjects who lost their sight at earlier developmental ages demonstrated a smaller GMV decrease in the visual areas. The interactions among developmental, plastic, and degenerative factors can also explain the alterations of GMV among different blind subgroups. Degenerative factors exert their effects on all blind subjects; thus, a common decreased GMV was identified in the visual areas, especially in the primary visual cortex [16, 44]. The developmental mechanisms play important roles in both the CB and EB, and the capacity for cross-modal plasticity is substantially stronger in the CB/EB than the LB; thus, “pruning interruption,” “rewiring,” and/or “unmasking” theories may take effect in the CB and EB, which can compensate for the degeneration-induced GMV reduction.

## 5. Conclusions

In summary, the present study demonstrated that the alterations of regional spontaneous brain activity and GMV are system-dependent, and an inverse association was identified between the regional spontaneous brain activity and GMV in blind individuals. Furthermore, the age of onset of blindness has different effects on the alteration of brain regional spontaneous brain activity and GMV. Our findings provided additional information regarding the alteration patterns of the spontaneous brain activity and their structural bases after visual deprivation at different developmental stages.

## Supplementary Material

The Supplementary Material provided the results about the influence of number of neighboring voxels on the ReHo measurements. ReHo values were calculated and compared using 7, 19, and 27 voxels neighboring criteria, respectively. First, we found a lower noise and higher contrast in ReHo map with higher voxel size (Supplementary Figure S1). Second, we found that similar *F* distributions of intergroup differences among the three datasets, and the dataset using 19 and 27 was more statistically significant than that using 7 voxels (Supplementary Figure S2).

## Figures and Tables

**Table 1:** Demographic information of the involved blind and sighted subjects.

Group	Gender	Age	Onset age
	(M/F)	Mean ± std.	Mean ± std.
CB	13/7	26.6 ± 5.0	0
EB	20/7	28.9 ± 7.4	8.0 ± 3.0
LB	30/14	30.9 ± 6.5	19.0 ± 5.3
SC	33/17	28.8 ± 7.00	/
Statistical value	*χ* ^2^ = 0.63	*F* = 2.07	*F* = 164.14
*P* value	0.889	0.107	<0.001

CB: congenitally blind, EB: early blind, and LB: late blind.
