# Supplementary material for: Alterations of Regional Spontaneous Brain Activity and Gray Matter Volume in the Blind
Source: Neural Plast. 2015 Oct 19;2015:141950. doi: 10.1155/2015/141950 (PMC4629052; doi:10.1155/2015/141950)
Supplement: Supplementary file 1 — The Supplementary Material provided the results about the influence of number of neighboring voxels on the ReHo measurements. ReHo values were calculated and compared using 7, 19, and 27 voxels neighboring criteria, respectively. First, we found a lower noise and higher contrast in ReHo map with higher voxel size (Supplementary Figure S1). Second, we found that similar F distributions of intergroup differences among the three datasets, and the dataset using 19 and 27 was more statistically significant than that using 7 voxels (Supplementary Figure S2). [file 141950.f1.doc]

Supplementary Results:

The influence of number of neighboring voxels on the ReHo measurements

Regional Homogeneity (ReHo) is used to measure the similarity (or Kendall’s coefficient concordance, KCC) of the time series of a given voxel to those of its directly neighboring voxels . The directly neighboring voxels can be defined by three criteria, face, edge and vertex, corresponding to 6, 18, and 26 voxels, respectively. In this study, to clarify if different neighboring voxels would influence the measurement of ReHo and group comparison in our study, we calculated the ReHo value using 7, 19, and 27 voxels (neighbors plus the voxel itself), respectively. We found a lower noise and higher contrast in ReHo map with higher voxel-size (Figure S1), indicating a better choice of ReHo with 27 voxels.


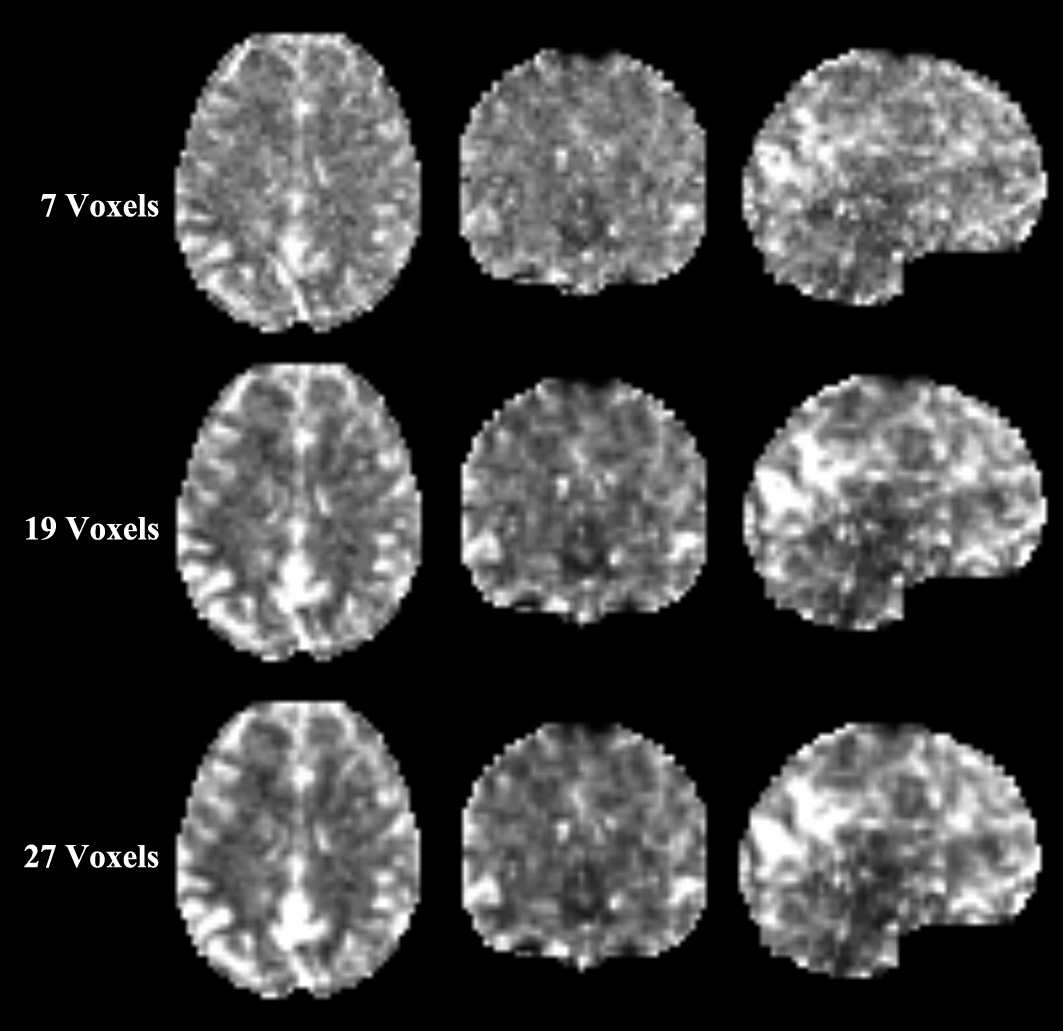


*Figure S1: The ReHo map calculated using different neighboring voxels of one subject. From the map we can see ReHo map with 7 voxels parameters is most noisy and has the lowest contrast in the default mode network (DMN), while ReHo map with 27 voxels is most clean and has the highest contrast in DMN.*

Furthermore, we compared the intergroup differences in ReHo values that were calculated using different neighboring voxels (One-way Analysis of Variance, P<0.05 corrected at the cluster level). As shown in Figure S2, the F distributions of intergroup differences were similar among these three dataset, and the dataset using 19 and 27 were more statistically significant in the middle cingulate cortex (MCC) than that using 7 voxels. Based on the above evidences, we chose the ReHo of 27 neighboring voxels as the main indices in this paper.


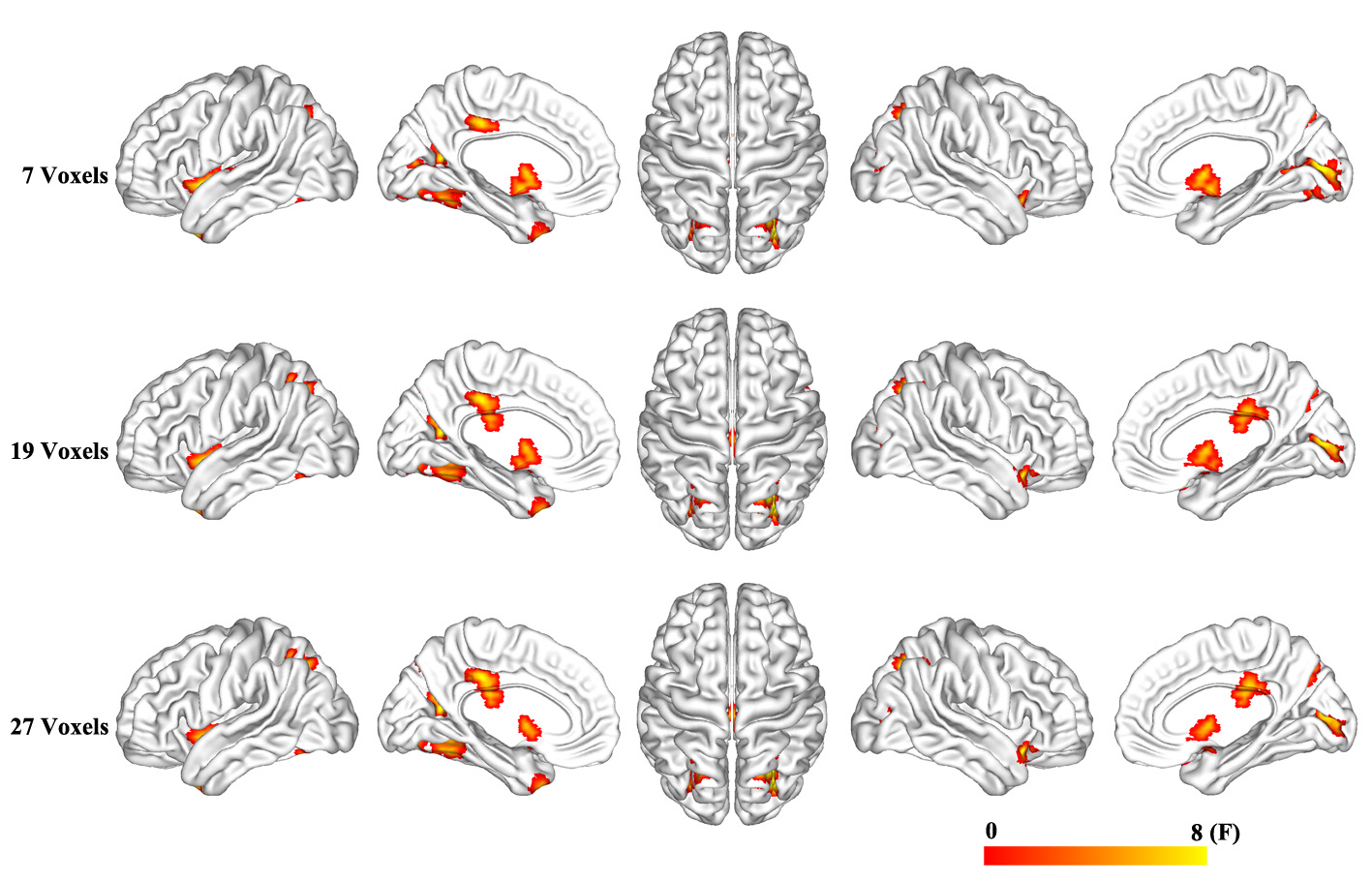


*Figure S2: F distributions of group differences in ReHo using different neighboring voxels among blind and sighted subjects. the F distributions of intergroup differences were similar among these three dataset, and the dataset using 19 and 27 were more significant in the middle cingulate cortex(MCC) than that using 7 voxels.*
